# All-cause mortality attributable to sitting time and physical inactivity in chilean adults

**DOI:** 10.1186/s12889-023-16467-0

**Published:** 2023-08-09

**Authors:** Ignacio Stingl-Zúñiga, Claudio Farías‑Valenzuela, Paloma Ferrero‑Hernández, Adilson Marques, Leandro F. M. Rezende, Antonio Castillo-Paredes, Carlos Cristi-Montero, Kabir P. Sadarangani, Gerson Ferrari

**Affiliations:** 1 Universidad de Santiago de Chile (USACH), Escuela de Ciencias de la Actividad Física, el Deporte y la Salud, Santiago, Chile; 2 Facultad de Ciencias Para El Cuidado de La Salud, Universidad San Sebastián, Lota 2465, 7510157 Providencia, Chile; 3 Escuela de Pedagogía en Educación Física, Facultad de Educación, Universidad Autónoma de Chile, 8900000 Santiago, Chile; 4 CIPER, Faculdade de Motricidade Humana, Universidade de Lisboa, Lisbon, Portugal; 5 Faculdade de Medicina, ISAMB, Universidade de Lisboa, Lisbon, Portugal; 6 Department of Preventive Medicine, Escola Paulista de Medicina, Universidade Federal de São Paulo, Sao Paulo, Brazil; 7 Grupo AFySE, Investigación en Actividad Física y Salud Escolar, Escuela de Pedagogía en Educación Física, Facultad de Educación, Universidad de Las Américas, Santiago, 8370040 Chile; 8 IRyS Group, Physical Education School, Pontificia Universidad Católica de Valparaíso, Valparaíso, Chile; 9 Universidad Autónoma de Chile, Santiago, Chile; 10 Escuela de Kinesiología, Facultad de Salud Y Odontología, Universidad Diego Portales, Santiago, Chile

**Keywords:** Non-communicable diseases mortality, Sedentary time, Sitting time, Physical inactivity, Mortality

## Abstract

**Background:**

Evidence on all-cause mortality attributable to joint sitting time and physical inactivity is lacking. In this study, we estimated the proportion and number of deaths attributable to sitting time and physical inactivity in Chilean adults.

**Methods:**

A sample of 5834 adults aged 20–96 years from a 2016–2017 Chilean National Health Survey was included to describe the prevalence of 16 joint categories of sitting time and physical activity. Relative risks for the joint association of sitting time and physical inactivity were obtained from a meta-analysis of individual participant data. We retrieved the number of deaths in adults ≥ 20 years in 2019 from the Chilean Ministry of Health.

**Results:**

Participants with high sitting time (> 8 h/day) and low physical activity (< 2.5 MET-hour/week) were more likely to be women, 20–64 years, non-indigenous ethnicity, lived in the urban areas, had middle education level and monthly household income, and had public health insurance. Reducing sitting time and increasing physical activity to a theoretical minimum risk exposure level could prevent up to 11,470 deaths or 10.4% of all deaths. Increasing physical activity to >35.5 MET-hour/week and maintaining sitting time could prevent approximately 10,477 deaths or 9.5% of all deaths. Reducing sitting time to < 4 h/day and maintaining physical activity would not reduce the number of deaths (-3.4% or 38 deaths).

**Conclusion:**

Reducing sitting time may be ancillary for preventing mortality. Therefore, increasing physical activity should be the primary focus of interventions and policies in Chile.

## Background

Epidemiologic studies have provided scientific evidence of the health benefits associated with moderate-to-vigorous intensity physical activity (MVPA) [1, 2]. Sedentary behavior refers to the lowest end of the physical activity spectrum, which has been defined as a low energy expenditure of < 1.5 metabolic equivalents (METs) in a sitting, lying or reclining posture during waking hours [3]. The links between sedentary behavior, chronic disease risk biomarkers, and all-cause and cause-specific mortality are not always well understood [4]. For instance, a systematic review and meta-analysis, based on 720,425 adults participants from nine prospective cohort studies including 25,769 cardiovascular events, demonstrated a nonlinear relationship between sitting time and risk of cardiovascular disease risk with increased risk only at very high sitting time (> 10 h/day), independent of physical activity level [5]. Another systematic review and meta-analysis examined the joint and stratified associations of sitting time and physical activity with all-cause mortality. Sitting time was associated with higher all-cause mortality, with association progressively attenuated with higher physical activity, to the point that association was nullified at 60–75 min/day of moderate- intensity physical activity [6].

Evidence-based guidelines are the basis of public health and clinical practice [7]. Considering the joint association of sitting time and physical activity, as well as the potential health benefits of various physical activity alternatives, is essential for expanding such recommendations. However, sedentary behavior guidelines are, at this time, nonspecific and are not always evidence-based [7, 8]. The World Health Organization 2020 guidelines on physical activity and sedentary behavior identified the lack of prospective studies on the joint effects of physical activity and sedentary behavior on all-cause mortality as major evidence gap [7].

Over the last decade, all-cause mortality attributable to sitting time or physical inactivity has been individually estimated in several countries for setting priorities for mortality prevention strategies [9–11]. A recent study estimated that increasing MVPA by 10, 20, or 30 min/day could potentially prevent 6.9%, 13.0%, and 16.9% of all deaths/year in US, respectively. Adding 10 min/day of physical activity resulted in an estimated 111,174 preventable deaths per year [9]. On the other hand, sitting time accounted for 3.8% (about 433,000 mortality/year) of all-cause mortality among 54 countries [10]. However, studies estimating all-cause mortality attributable to joint sitting time and physical inactivity are lacking.

Currently, 27% of the Chilean population is physical inactivity, and 38% spend ≥ 4 h/day sitting. Despite regular national surveys on the prevalence of sitting time and physical inactivity, evidence on all-cause mortality attributable to sitting time and physical inactivity is lacking in the country. Such information could inform future public health policies and interventions to reduce sitting time and improve physical activity. In this study, we aimed to estimate the proportion and number of deaths attributable to joint sitting time and physical inactivity in Chilean adults by using national representative data, Official Deaths Statistics and results from a meta-analysis.

## Methods

We developed a macrosimulation model to estimate the all-cause mortality attributable to joint sitting time and physical inactivity in Chilean adults. The modeling approach involved three stages: (1) estimating the prevalence of joint categories of sitting time and physical activity using nationally representative survey data of Chilean adults, (2) establishing the counterfactual scenarios of reducing sitting time and increasing physical activity, and (3) estimating the effect of reduced sitting time and physical inactivity via comparative risk assessment analysis.

### Assessment of sitting time and physical inactivity

We obtained sitting time, and physical inactivity from the Chilean National Health Survey (*Encuesta Nacional de Salud – NHS*) conducted between 2016–2017. The NHS is a representative survey, complex, random, stratified, and multistage probability sampling strategy, including 6233 participants aged ≥ 15 years from urban and rural areas in Chile. The protocol of each wave of the NHS 2016–2017 was approved by the Ethics Committee of the Pontificia Universidad Católica de Chile (Pontifical Catholic University of Chile—(No. 16–019), institution in charge of the studies. A statement to confirm that all methods were carried out in accordance with relevant guidelines and regulations. Participants signed an informed consent to take part in the study. A detailed description of the sampling process is available elsewhere [12]. In this study, we excluded participants < 20 years old and participants with missing data of sitting time or physical inactivity (*n* = 399). Our final analytical sample included data from 5834 adults aged ≥ 20 years with complete data for sitting time and physical activity.

The Global Physical Activity Questionnaire (GPAQ) was used to assess sitting time and physical activity. Participants reported the duration, frequency, and intensity of physical activities performed in three different domains (occupational, active commuting, and recreational), comprising 16 questions, including one about sitting time [13, 14]. Total self-reported physical activity was calculated as the sum of MET-hour/week across all three domains. Participants were subsequently categorized into < 2.5 MET-hour/week, 2.5–16.0 MET-hour/week, 16.1–30.0 MET-hour/week, and > 30.0 MET-hour/week [6]. The median time spent in physical activity was 315.0 (interquartile range [IQR]: 1.5; 680.0) min/week. Sitting time was derived using the following question: how much time do you usually spend sitting or reclining on a typical day? [15]. We categorized sitting time data in four groups (0– < 4 h/day, 4– < 6 h/day, 6–8 h/day, and > 8 h/day) [6]. The median time spent in sitting was 120 (interquartile range—IQR: 60.0; 240.0) min/day. Finally, we jointly categorized participants into 16 categories of sitting time and physical activity by using the categories described above.

### All-cause mortality: relative risks and number of deaths

We retrieved the number of deaths in Chile in 2018 from the Official Deaths Statistics of the Ministry of Health [16, 17]. Relative risks (RR) and 95% confidence intervals (CI) for the joint association of sitting time and physical inactivity (reference group: 0– < 4 h/day of sitting time and > 30.0 MET-hour-week of physical activity) for all-cause mortality were retrieved from a meta-analysis of individual participant data [6].

### Statistical analysis

As appropriate, median (and IQR) and percentages were computed in descriptive analysis. Statistical analyses were performed using the software IBM SPSS, v.26. (SPSS Inc., IBM Corp., Armonk, New York, NY, USA) and NHS complex sampling strategy was taken into account.

We estimated the prevalence of the joint categories of sitting time and physical activity (Table 1). Then, we calculated the potential impact fraction (PIF) for all-cause mortality using the following equation:

$$PIF=\frac{{\sum\nolimits}_{i=1}^{n}{P}_{i}\;{RR}_{i}- {\sum\nolimits}_{i=1}^{n}{P}_{i}^{\prime}{RR}_{i}}{{\sum\nolimits}_{i=1}^{n}{P}_{i}\;{RR}_{i}}$$

where P is the prevalence of joint categories of sitting time and physical activity, P* is the counterfactual scenario of reducing sitting time and increasing physical activity, and RR is the relative risk for all-cause mortality. We considered three counterfactual scenarios: Scenario 1: theoretical minimum risk exposure level, where all participants are in the low sitting time (0– < 4 h/day) and high physical activity (> 30.0 MET-hour/week) categories; 2) Scenario 2: increasing physical activity to > 30.0 MET-hour/week and maintaining sitting time level; Scenario 3: maintaining physical activity level and decreasing sitting time to 0– < 4 h/day. To obtain the number of deaths attributable to joint sitting time and physical inactivity, we applied PIF to total number of deaths that occurred in Chile in 2018.

**Table 1:** Participants characteristics according to the extreme joint categories of sitting time and physical inactivity in Chilean adults

Variables	Total (*n* = 5834)	Low sitting time and high physical activity (*n* = 1140)	High sitting time and low physical activity (*n* = 243)
Sex			
Men	36.4	48.4	36.6
Women	63.6	51.6	63.4
Age group			
Adults (20–64 years)	74.0	85.4	61.3
Older adults (≥ 65 years)	26.0	14.6	38.7
Region of Chile			
North	26.7	22.3	27.2
Center	47.4	50.8	50.2
South	25.9	26.9	22.6
Geographic area			
Urban	83.9	80.3	93.8
Rural	16.1	19.7	6.2
Education level			
< 8 years	25.3	22.9	35.1
8–12 years	51.7	58.2	37.7
> 12 years	22.9	18.9	27.2
Monthly household income			
Low	26.2	20.1	22.7
Middle	49.4	56.2	46.8
High	24.4	23.7	30.5
Health insurance			
Public	84.1	85.7	79.1
Private	11.8	8.6	17.9
Other/none	4.1	5.7	3.0
Indigenous ethnicity			
Yes	11.2	12.0	5.8
No	88.8	88.0	94.2

Low sitting time: 0–4 h/day; High sitting time: > 8 h/day;

Low physical activity: quartile 1; High physical activity: quartile 4

## Results

A total of 5834 adults (63.6% women) with an average age of 51.1 (standard deviation: 18.0) participated in the study. Overall, 74.0% were between 20–64 years of age, 47.4% were from the center region, 83.9% lived in urban areas, 51.7% had between 8 to 12 years of educational level, 49.4% had a low monthly income, 84.1% had public health insurance, and 88.8% did not belong to any indigenous ethnic group (Table 1).

Table 1 presents the characteristics of the participants according to the two extreme categories of physical activity and sitting time (i.e., low sitting time [0–4 h/day] and high physical activity [> 30.0 MET-hour/week] vs high sitting time [> 8 h/day] and low physical activity [< 2.5 MET-hour/week]). Participants with high sitting time and low physical activity were more likely women, adults aged 20–64 years, non-indigenous ethnicity, lived in the North region and urban areas, had higher education level and monthly household income, and were more likely to have private health insurance than participants with low sitting time and high physical activity (Table 1).

**Fig. 1:**
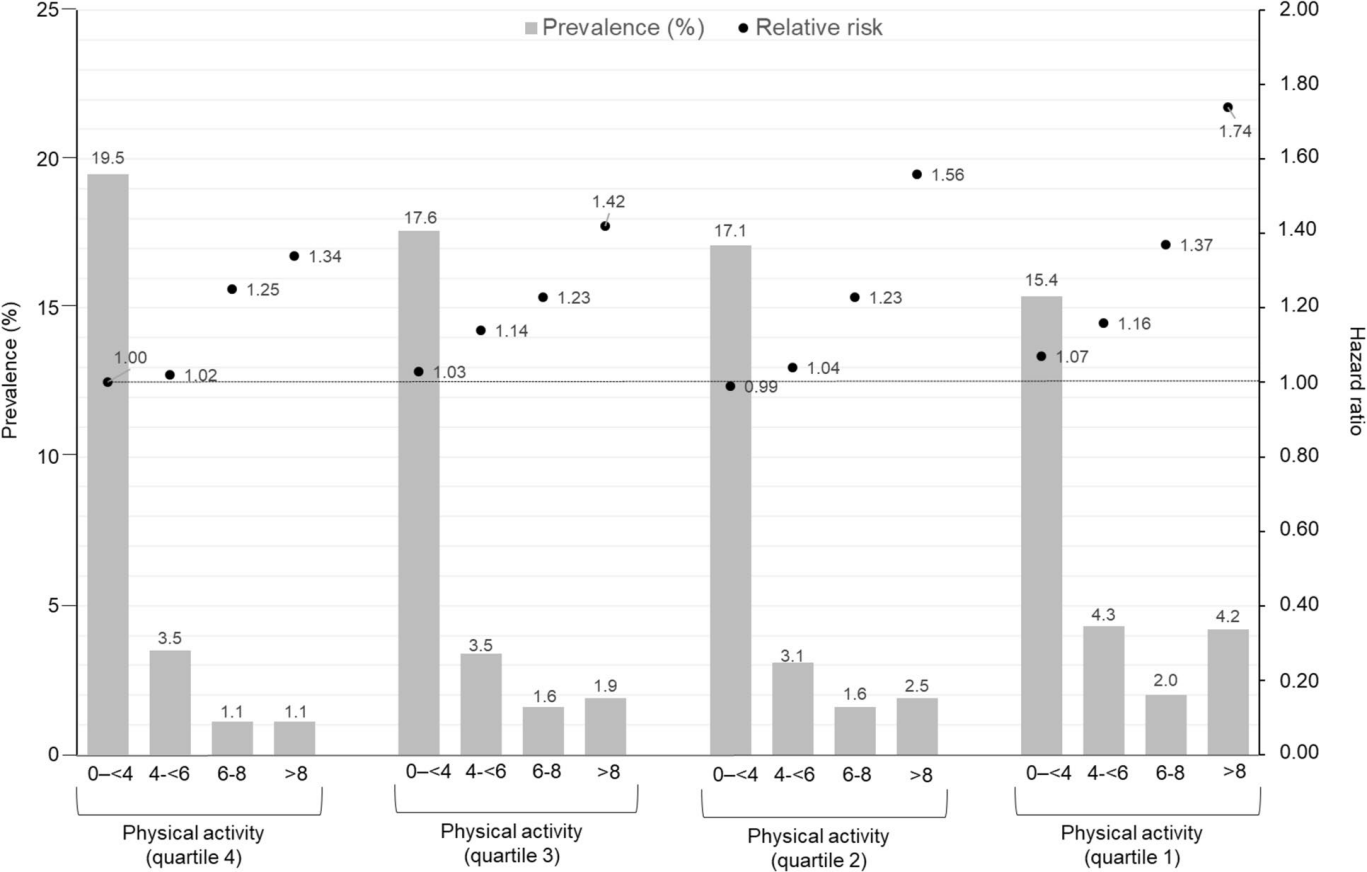
Prevalence (%)of sitting time and physical activity categories in adults from Chile and relative risks for all-cause mortality [6]

Figure 1 shows the proportion of adult’s participants in each of the 16 categories of physical activity and sitting time. The proportion of participants in the two extreme categories of physical activity and sitting time were 19.5% (low sitting time [0–4 h/day] and high physical activity [> 30.0 MET-hour/week]) and 4.2% for high sitting time [> 8 h/day] and low physical activity [< 2.5 MET-hour/week]), respectively.

**Fig. 2:**
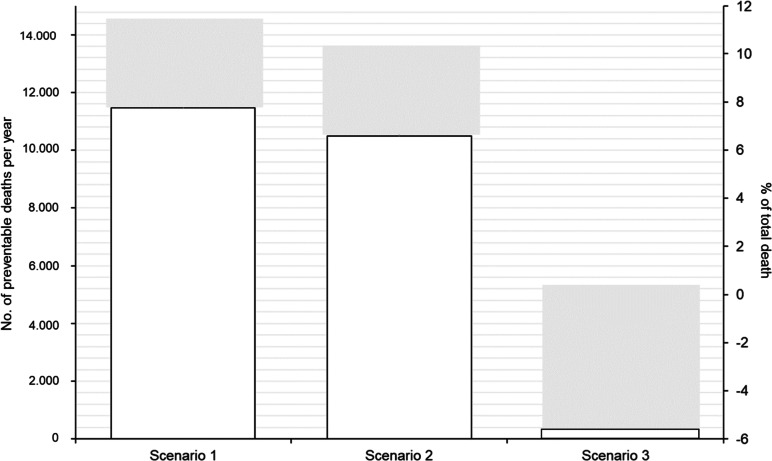
Number and proportion of preventable deaths per year in adults from Chile by counterfactual scenario. Scenario 1: all participants in the low sitting time (0– < 4 h/day) and high physical activity (> 30.0 MET-hour/week); Scenario 2: increasing physical activity to > 30.0 MET-hour/week and maintaining the level of sitting time; Scenario 3: maintaining physical activity level and decreasing sitting time to 0– < 4 h/day

We estimated that reducing sitting time and increasing physical activity to a theoretical minimum risk exposure level (Scenario 1: < 4 h/day of sitting and > 30.0 MET-hour/week of physical activity) could prevent 11,470 deaths or 10.4% of all deaths in Chile in 2019. Increasing physical activity to > 30.0 MET-hour/week and maintaining sitting time could prevent, approximately, 10,477 or 9.5% of all deaths. On the other hand, reducing sitting time to < 4 h/day and maintaining physical activity level would not reduce the number of deaths (-3.4% or 37.5 deaths) (Fig. 2).

## Discussion

In this study, we estimated the number and proportion of deaths attributable to joint sitting time and physical inactivity in Chilean adults. We found that increasing physical activity and reducing sitting time to a theoretical minimum risk exposure level (< 4 h/day of sitting time and > 30.0 MET-hour/week) could prevent 11,470 deaths in Chile in 2019. Increasing physical activity to > 35.5 MET-hour/week and maintaining sitting time could prevent approximately 10,477 deaths. Reducing sitting time to < 4 h/day and maintaining physical activity would not reduce the number of deaths (38 deaths).

The joint effect of physical activity and sitting time on mortality has received great attention in the literature [6, 18, 19]. Early judgements recommended that the effects of sedentary behavior were mainly independent of physical activity, given that the relations remained substantial even after adjustment by MVPA [20]. Conversely, epidemiologic data most accepted nowadays suggests that physical activity is an effect modifier for the association between sedentary behavior and mortality, such that the detrimental effects of excessive sedentary behavior are more pronounced in physically inactive persons [21], because the latter do not comply with the physical activity recommendations and spend more time in sedentary activities [22]. For instance, a meta-analysis of individual data revealed that the effect of sedentary time on all-cause mortality was greater among those with lower levels of physical compared with those with higher levels of physical activity (HR: 1.46 vs 1.16, respectively) [23]. In addition, and in agreement with our results, the combined association between higher levels of physical inactivity and a longer time spent in sedentary behavior increased the risk of mortality compared to the most active groups in older adults [24].

A systematic review and meta-analysis of data from more than 1 million adults estimated the joint effects of sitting time and MVPA on the risk of cardiovascular disease mortality [6], showing that within each level of daily sitting, there is an opposite graded dose–response association between MVPA and cardiovascular disease mortality. Of note, even among those sitting > 8 h/day, the HR for those undertaking  > 30.0 MET-hour/week of physical activity (approximately 60–75 min/day of MVPA) was not significantly elevated compared to those sitting < 4 h/day. This recommends that the detrimental effects of sitting time on cardiovascular disease mortality may be eliminated by undertaking high levels of physical activity, approximately, 60–75 min/day. Therefore, actions based on promoting compliance with the physical activity recommendations and less sedentary time in combination, should be considered together, given the inverse association with the risk of mortality, whose relationship is independent of the nutritional status determined by the body mass index [25].

Our results support continued efforts to promote physical activity in those segments of the physically inactive inhabitants. Lower daily sitting time reduced the risk among those who reported > 8 h of sitting/day. However, risks remained considerably elevated compared with the reference group, who were highly active and sat for < 4 h/day. Such findings suggest that reducing sitting times may be insufficient for optimal health benefit in the absence of some physical activity. Considering it might be challenging to achieve high levels of physical activity for physically inactive people, particularly for adults, reducing sitting time might be an essential first step toward improving health. The second step would be increasing daily life activities intensity, [7] as well as the implementation of actions that economically encourage the reduction of physical inactivity and sitting time [26]. Third, the promotion of physical activity and the reduction of sedentary behaviors should be established from school stages to the formation of habits for better health from childhood and adolescence [27] and, in this way, reducing sitting time and promote the practice of MVPA, which are reduced as the years go by [28]. Finally, compliance with higher levels of physical activity, regardless of its intensity, with a parallel reduction in the time dedicated to sedentary behaviors, contribute directly to premature mortality rates, with an interdependence in the dose–response [29].

Our study has several limitations. First, high-quality, long-term prospective cohort studies on sitting time, physical inactivity, and mortality are inexistent in Chile. Therefore, we used RR from a meta-analysis of individual participant data from more than one million men and women enrolled in prospective cohort studies from other high-income countries (Canada, Australia, United States, and European countries). Whether these RR apply to the Chilean population is unknown and warrants further investigation. We did not consider potential effect modifiers, such as sex, age, ethnicity, and socioeconomic status. A similar methodological approach has been used in previous country-wide PAF estimates in the United States [30], Argentina [31], and Brazil [32]. Second, we used the validated [13, 14] but self-reported sitting time and physical activity data from 2016–2017, the most recent nationally representative survey was conducted in Chile. This may have introduced misclassification bias due to errors inherent to questionnaires and changes in sitting time and physical activity over time.

## Conclusion

We found that joint sitting time and physical inactivity may be responsible for 10% of all deaths in Chile. These findings support implementing evidence-based strategies to increase physical activity and reduce sitting time in Chilean adults to reduce all-cause mortality. Increasing physical activity should be the primary focus of these interventions and policies, whereas reducing sitting time may be ancillary to reducing mortality.

## Data Availability

Data may be obtained from a third party and are publicly available. This study is based in part on data from Chilean National Health Survey and Chilean Ministry of Health. More information is available on the website: http://epi.minsal.cl/encuesta-ens-descargable/. For further information, please contact the corresponding authors in the first instance.
